# Supplementary material for: Foam film vitrification for cryo-EM
Source: Nat Commun. 2025 Jul 4;16:6199. doi: 10.1038/s41467-025-61270-7 (PMC12227703; doi:10.1038/s41467-025-61270-7)
Supplement: Supplementary file 3 — Description of Additional Supplementary Files [file 41467_2025_61270_MOESM3_ESM.pdf]

Description of Additional Supplementary Files

File Name: Supplementary Movie 1
Description: Reconstructed tomograms of catalase at a concentration of 6 g L-1with 0.01% DDM, prepared using the foam film method. Tilt series were recorded at nominal magnifications of ×53,000, with calibrated pixel sizes of 2.39 Å.

File Name: Supplementary Movie 2
Description: Reconstructed tomograms of catalase at a concentration of 0.1 g L-1, prepared using the Vitrobot without surfactant. Tilt series were recorded at nominal magnifications of ×53,000, with calibrated pixel sizes of 2.39 Å.

File Name: Supplementary Movie 3
Description: Reconstructed tomograms of catalase at a concentration of 0.5 g L-1 with 0.01% DDM, prepared using the Vitrobot. Tilt series were recorded at nominal magnifications of ×53,000, with calibrated pixel sizes of 2.39 Å.

File Name: Supplementary Movie 4
Description: Reconstructed tomograms of 30S ribosomes at a concentration of 3.4 g L-1 with 0.01% DDM, prepared using foam film method. Tilt series were recorded at nominal magnifications of ×81,000, with calibrated pixel sizes of 1.514 Å.

File Name: Supplementary Movie 5
Description: Reconstructed tomograms of 30S ribosomes at a concentration of 3.4 g L-1, prepared using the Vitrobot without surfactant. Tilt series were recorded at nominal magnifications of ×81,000, with calibrated pixel sizes of 1.514 Å.

File Name: Supplementary Movie 6
Description: Reconstructed tomograms of 30S ribosomes at a concentration of 3.4 g L-1 with 0.01% DDM, prepared using the Vitrobot. Tilt series were recorded at nominal magnifications of ×53,000, with calibrated pixel sizes of 2.39 Å.

File Name: Supplementary Movie 7
Description: Reconstructed tomograms of EspB at a concentration of 5 g L-1 with 0.01% DDM, prepared using foam film method. Tilt series were recorded at nominal magnifications of ×81,000, with calibrated pixel sizes of 1.514 Å.

File Name: Supplementary Movie 8
Description: Reconstructed tomograms of EspB at a concentration of 1 g L-1, prepared using the Vitrobot without surfactant. Tilt series were recorded at nominal magnifications of ×81,000, with calibrated pixel sizes of 1.514 Å.

File Name: Supplementary Movie 9
Description: Reconstructed tomograms of EspB at a concentration of 3 g L-1 with 0.01% DDM, prepared using the Vitrobot. Tilt series were recorded at nominal magnifications of ×53,000, with calibrated pixel sizes of 2.39 Å.

File Name: Supplementary Movie 10

Description: Reconstructed tomograms of EspB 14-mers at a concentration of 2 g L-1with 0.67% DDM, prepared using foam film method. Tilt series were recorded at nominal magnifications of ×81,000, with calibrated pixel sizes of 1.514 Å.

File Name: Supplementary Movie 11
Description: Reconstructed tomograms of EspB 14-mers at a concentration of 2 g L-1with 0.67% DDM, prepared using the Vitrobot. Tilt series were recorded at nominal magnifications of ×53,000, with calibrated pixel sizes of 2.39 Å.

File Name: Supplementary Movie 12
Description: Reconstructed tomograms of 20S proteasomes at a concentration of 0.7 g L-1 with 0.01% DDM, prepared using foam film method. Tilt series were recorded at nominal magnifications of ×64,000, with calibrated pixel sizes of 1.38 Å.

File Name: Supplementary Movie 13
Description: Reconstructed tomograms of 20S proteasomes at a concentration of 0.7 g L-1, prepared using the Vitrobot without surfactant. Tilt series were recorded at nominal magnifications of ×81,000, with calibrated pixel sizes of 1.514 Å.

File Name: Supplementary Movie 14
Description: Reconstructed tomograms of AcrB at a concentration of 3 g L-1 with 0.03% DDM, prepared using foam film method. Tilt series were recorded at nominal magnifications of ×81,000, with calibrated pixel sizes of 1.514 Å.

File Name: Supplementary Movie 15
Description: Reconstructed tomograms of AcrB at a concentration of 3 g L-1 with 0.03% DDM, prepared using the Vitrobot. Tilt series were recorded at nominal magnifications of ×53,000, with calibrated pixel sizes of 2.39 Å.
